# Supplementary material for: Hepatoprotective Effects of Four Brazilian Savanna Species on Acetaminophen-Induced Hepatotoxicity in HepG2 Cells
Source: Plants (Basel). 2023 Sep 26;12(19):3393. doi: 10.3390/plants12193393 (PMC10574628; doi:10.3390/plants12193393)
Supplement: Supplementary file 1 [file plants-12-03393-s001.zip › plants-2592567-supplementary.pdf]

Supplementary material

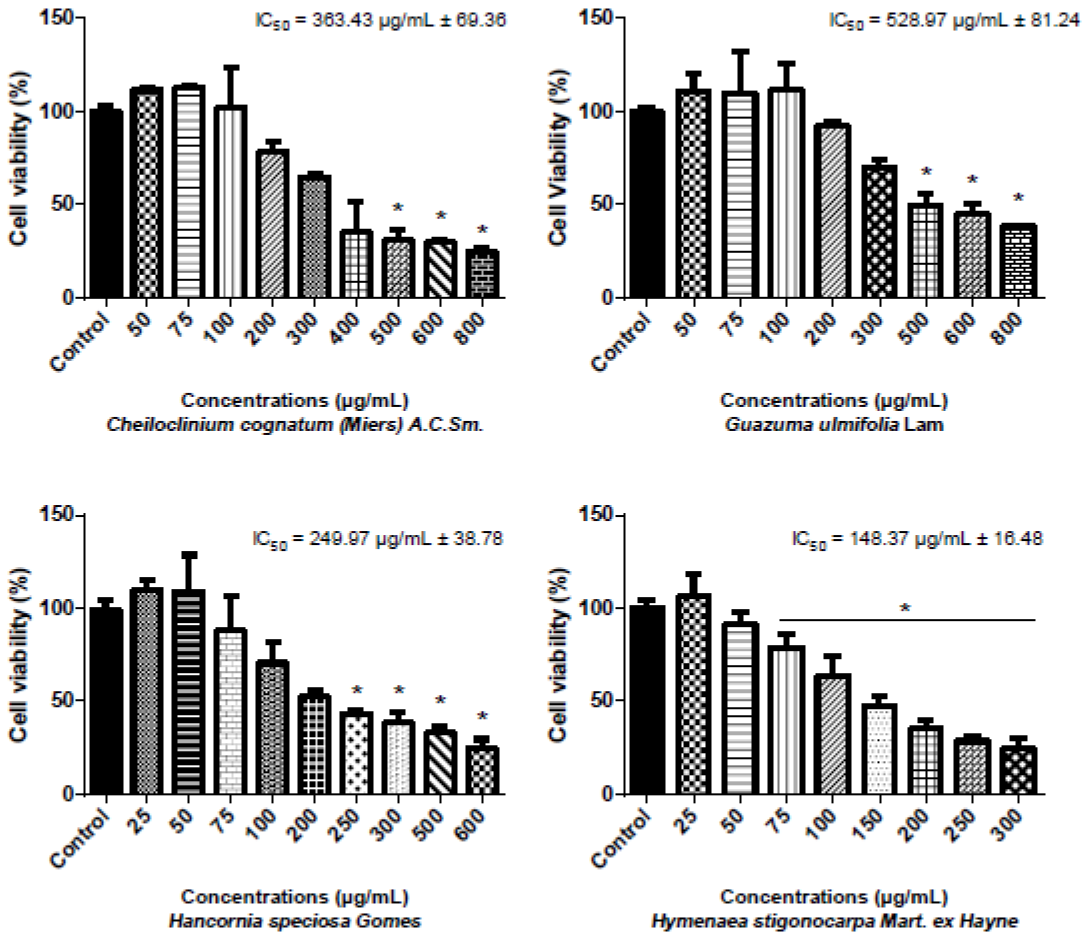

Figure S1. HepG2 cell viability after 24 hours of treatment with the aqueous extract of *Cheiloclinium cognatum* (Miers) A.C.Sm, *Guazuma ulmifolia* Lam., *Hancornia speciosa* Gomes and *Hymenaea stigonocarpa* leaves. Cell viability values (%) are expressed as a median and interquartile range (n=3). The viability results of the different extract concentrations were compared with the control by Kruskal-Wallis with Dunn's post-test (* statistical difference from the control, $p \leq 0.05$).

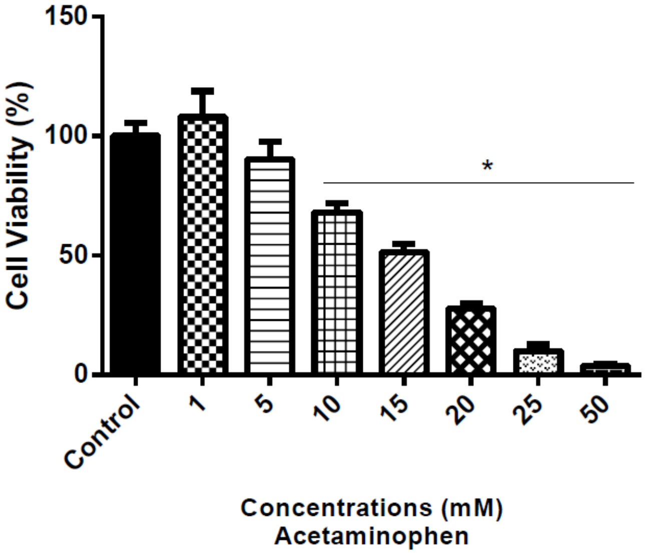

Figure S2. Cellular viability of HepG2 after 24 hours of treatment with acetaminophen**.** Cell viability values (%) are expressed as mean and standard deviation (n=3). The viability results of different paracetamol concentrations were compared with the control by analysis of variance (ANOVA) with Dunnet post-test. (*$p \leq 0.05$).

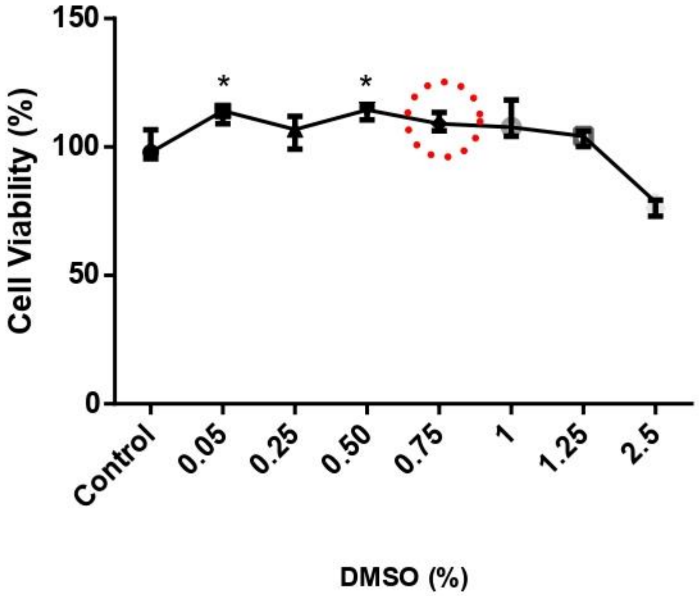

Figure S3. HepG2 cell viability after 24 hours of treatment with DMSO concentrations used to prepare the APAP concentrations. The 0.05% concentration corresponds to the lowest concentration on the APAP curve (1 mmol/L) and the 2.5% concentration corresponds to the highest concentration on the APAP curve (50 mmol/L). The concentration in the graph corresponds to the percentage of DMSO used to prepare the 15 mmol/L concentration used in the hepatoprotection assay. Cell viability values (%) are expressed as the median and interquartile range (n=3). The viability results of different extract concentrations were compared with control by Kruskal-Wallis with Dunn's post-test (*, $p \leq 0,05$).

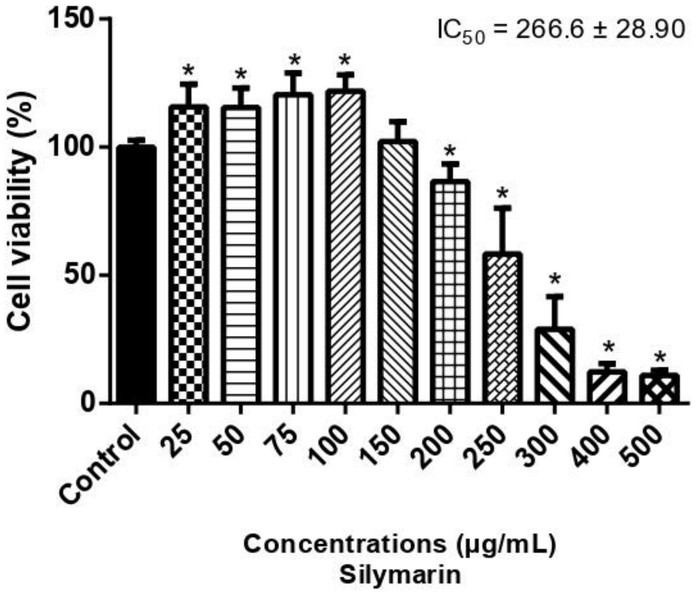

Figure S4. HepG2 cell viability after 24 hours of treatment with the concentrations of silymarin. Cell viability values (%) are expressed as mean and standard deviation (n=3). Viability results of different concentrations of the compound were compared with the control by analysis of variance (ANOVA) with Dunnet's post-test. (*, p ≤ 0,05).
